# Deep venous stenting in females

**DOI:** 10.1186/s42155-023-00354-1

**Published:** 2023-03-15

**Authors:** Gerry O’Sullivan, Steven Smith

**Affiliations:** 1 Interventional Radiology, Galway University Hospitals, Galway, Ireland; 2 Chicago, USA

## Abstract

Deep venous stenting has gained increasing prominence in recent years. This issue focuses on special considerations in female patients. The specific challenge relates to the fact that these patients are often much younger than those in whom arterial stents are placed. The stents have to perform adequately over potentially 60 years- and there is no data of that length available.

## Introduction

Indications for venous stenting include: Table 1 (Breen, 2020; de Graaf et al., 2013; Mussa et al., 2007; Hartung et al., 2009):

1. Acute and chronic venous obstruction.
2. To alleviate symptoms of pelvic venous obstructive disease.
3. In the treatment of venous stenosis in patients with recurrent lower extremity varicose veins.

**Table 1:** Lower extremity venous stenting indications

Clinical condition	Clinical findings	Imaging	Recommendation	Reference
Acute IF DVT Ilio-Femoral Deep Vein Thrombosis	Swollen tense leg	IF DVT +	Thrombectomy/Stent	CLEAR DVT
		Caus. stenosis		(Contemporary Endovascular Therapies in Treatment of Acute Iliofemoral Deep Vein Thrombosis (CLEAR-DVT), n.d.)
Acute IF DVT	Minimal	IF DVT+/-	Anticoagulation only	ATTRACT
		Caus. stenosis		(Vedantham et al., 2017)
PTS Post Thrombotic Syndrome	Leg ulcer	Scarred/Occl iliac v.	Stent	(de Graaf et al., 2013; Mussa et al., 2007; Hartung et al., 2009)
PTS	Minimal swelling	Scarred/Occl iliac v.	Stockings Anticoagulation	(Raju et al., 2002)
Pelvic Vein Congestion	Minimal	Stenosis L CIV	Assess degree of stenosis	Pappas (Sulakvelidze et al., 2021)
Rapidly recurrent Leg Varices	Obvious varicose Veins	Stenosis CIV	Assess degree of stenosis; +/- stent	Raju (2002)
HfPEF Heart Failure Preserved Ejection Fraction	Shortness of Breath	IVC occlusion	IVC stent	Morris (Morris et al., 2020)
Cancer	Swollen Legs	Lymph nodes	Stent	(O'Sullivan et al., 2015)
“Lymphoedema”	Swollen Legs	all normal	Manual Lymphatic Drainage	Gasparis (Gasparis et al., 2020)

Acute and chronic venous obstruction have been extensively dealt with elsewhere, but deserve some mention here (Mahnken et al., 2014; Seager et al., 2016; Editor's Choice – European Society for Vascular Surgery (ESVS), 2022; O’Sullivan, n.d.). Females of childbearing years are at increased risk of venous thrombo-embolic disease, not just from pregnancy and the puerperium, but also from the oral contraceptive pill (OCP). OCP is usually regarded as a “permissive” rather than a “causative” factor (de Bastos et al., 2014). A common combination appears to be the OCP and a stenotic lesion e.g. Iliac Vein Compression Syndrome (May-Thurner) (Narayan et al., 2012; O'Sullivan et al., 2000). Once the thrombus has been dissolved/removed, an underlying stenotic lesion is frequently revealed. The likelihood of finding a lesion depends on the intensity and accuracy of the search for it; so good quality CTV or MRV pre op and IVUS intra-op (see below) are the preferred options (O’Halloran N, Lehane C, O. Malley E, O’Sullivan GJ. (n.d.) Iliac vein lesions are frequently missed by radiologists on cross sectional imaging leading to delays in diagnosis. (CVIR submitted)). Assuming a lesion is found then it needs to be dealt with by means of a stent. Angioplasty on its own is rarely sufficient (Patel et al., 2000).

The remainder of the article deals with the other patient groups. Pelvic venous disease in women has attracted increasing significance (Bałabuszek et al., 2022; Sulakvelidze et al., 2021; Tanaka et al., 2021), and a proportion of these patients have an underlying deep venous stenosis. Venous stents may be indicated in some patients. However, this area is contentious as there are no hard and fast diagnostic rules to decide which patients merit stent placement. Many of these patients are young, and the stent must stay open, in position, and not fracture- for up to 50 years.

### Diagnostic methods, pre-operative approach

**Fig. 1:**
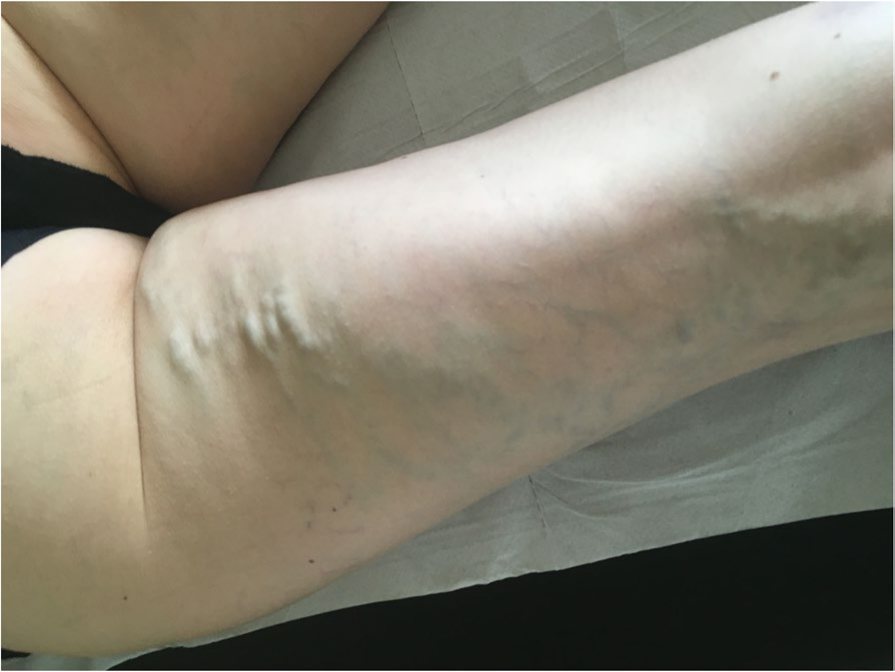
Posterior thigh varices should raise suspicion of a pelvic source. Prone view of a patient with a characteristic pattern

A thorough, detailed history and focused physical examination are mandatory before considering venous stent placement (Tanaka et al., 2021). Accurate cross-sectional imaging is also essential to improve patient selection. Practitioners can employ Ultrasound, CT venography, and MR venography as initial diagnostic methods (Zucker et al., 2016; Coelho & O'Sullivan, 2019; Coelho & O'Sullivan, 2020). Focused questions are required on walking distance, presence or absence of venous claudication, weight gain, gravitational pain (pain which is worse in the evenings rather than the mornings), post-coital pain (not occurring immediately but it can last for several hours after coitus), urinary symptoms, the number of pregnancies, recurrent spontaneous abortion, use of the oral contraceptive pill and occasionally, irritable bowel type symptoms (Tanaka et al., 2021; Khilnani, 2022; Latthe et al., 2006; Leatherby et al., 2020; Meissner et al., 2021; Beard et al., 1988). The pain is usually absent upon awakening, but starts with upright activity in the morning. Although it is often characterized as “non-cyclic pain”, it may worsen around ovulation and menses to a variable extent. Perform the initial clinical examination with the patient standing. With appropriate consent, photographs of external sequelae, including venous hypertensive change, varicose veins, vulvar varicosities, or venous ulceration, are helpful. Lower extremity varicose veins which occur in an atypical pattern should alert the physician to the possibility of a pelvic source (Fig. 1- posterior thigh varices- taken with patient permission).

Often patients are referred by a gynaecologist or urologist; their imaging will reflect this; if they have seen a gynaecologist, they will usually have undergone either a transabdominal or transvaginal ultrasound which may demonstrate pelvic varices. Occasionally the varices will have been demonstrated on laparoscopy, but a negative laparoscopy report is not helpful (Steenbeek et al., 2018; Baekgaard et al., 2017).

Depending on availability, either a non-oral contrast post IV contrast-enhanced CT abdomen and pelvis to below the lesser trochanters or combined MRA/MR venography is performed; the latter is preferable as it does not employ radiation nor intravenous contrast (Baekgaard et al., 2017). MRA is necessary as late arterial shunting of blood is seen patients with relux; standard MRV may miss this. In addition, using phase-encoded MRV, the flow direction, specifically in the gonadal veins, can be evaluated; we have found this to be very helpful in identifying which veins are likely to require embolization (Dick et al., 2010; Meneses et al., 2011; Asciutto et al., 2008).

Unlike in arterial disease, there are no set criteria for identifying which patients have a causative venous stenosis on MRV/CTV. Normal ranges have been established (Arendt et al., 2020), but these suffer from a lack of uniformity of the degree of pre-scan hydration, and whether the scan is performed on inspiration, expiration, or Valsalva. This lack of standardisation may have indirectly led to the recent increase in venous stent insertion and subsequent migration (Sayed et al., 2022); or in the case of dehydration before scanning, a false negative scan and potential under-treatment.

Pressure measurements are of proven benefit in arterial disease (Kinney & Rose, 1996) but less so in deep venous (Mahnken et al., 2014). The gradients are small, and the real issue is the ambulatory venous pressure differential (Kurstjens et al., 2016) as opposed to recumbent non-ambulatory venous pressures. 

### Intraoperative imaging

Regardless of the site of access to the deep venous system (internal jugular, femoral, etc.), a combination of venography and intravascular ultrasound is ideal for evaluating the deep veins. Intravascular ultrasound is of proven benefit in more accurate identification of lesions which are missed on venography. Venography is better at demonstrating collaterals, the flow velocity, and the rate of contrast washout; however, it is less sensitive to identifying stenosis, particularly smooth, gradual stenosis rather than abrupt focal lesions (Neglén & Raju, 2002; McLafferty, 2012; Montminy et al., 2019; Gagne et al., 2018). Fasting patients are all relatively dehydrated, and this may flatten the “reference segment” of the iliac vein, and lead to calculating falsely low percentage stenosis on IVUS. These patients are best served by 2 l 0.9 NACL IV pre procedure and continued hydration support (author’s experience SJS).

Ideally both venography and intravascular ultrasound are performed on every patient. There is currently no randomised control trial to demonstrate which is more likely to yield a successful outcome in *pelvic venous disorders*. Although IVUS is a very accurate method of identifying venous stenosis, a significant proportion of our readers may not have access to it. IVUS has been approved for coverage in the United States, but this has not been extended worldwide. The cost of the console and catheters is a factor. An alternative (but again, one without a randomised control trial), is the passage of a gently inflated (< 1 atm) 14 mm balloon through the iliac veins; if no “catch” or hold up to the passage of balloon is felt, it is unlikely that there is a significant stenosis. Therefore, a combination of these three methods may be employed to verify the existence of venous stenosis.

### Venous stents specifically in pelvic venous disorders

Patients with both pelvic vein congestion syndrome, and those who develop recurrent varicose veins may have an underlying non-thrombotic iliac vein lesion (NIVL). It is essential to identify those patients who will benefit from venous stenting. There has been a scarcely credible rise in the performance of venous stenting, and it is difficult to believe that all of it is clinically justified. Patients may have a combination of venous stenosis (in the renal vein or common iliac vein), and reflux (into the gonadal veins and internal iliac veins). Which procedure should you perform first; and in what order? Some experts have shown benefit from iliac vein stent placement on its own, while others have shown that pelvic vein embolisation alone results in a high proportion of clinical relief (De Gregorio et al., 2020; Lakhanpal et al., 2021; Harris, 2021). A variety of factors may be at play here; including the age of the patient, their gravid status, and whether they also have varicose veins in the legs (Sulakvelidze et al., 2021).

Based on fairly large series, it appears that both iliac vein stent placement and pelvic vein embolization performed individually, have no effect on subsequent pregnancy rates (Liu et al., 2019; Dos Santos et al., 2017). There is no data to confirm that this is the case if performed concurrently. In patient with both reflux and iliac vein obstruction, and no desire to retain fertility, then performing pelvic vein embolisation and venous stent placement at the same time, makes sense. In a patient who may wish to become pregnant subsequently there is no data to support which treatment should be performed first, or whether they should be performed together. Therefore, patients need to have appropriately consented before the procedure. It will also change the post-operative management as the patient will likely require some degree of anticoagulation if a venous stent is placed. Finally, venous stent placement typically causes low back pain for a variable duration (Snow et al., 2023), and this may affect the type of anticoagulation used as non-steroidal medications may interact with both Warfarin and newer oral anticoagulant (NOACS).

Again, it must be borne in mind that many of these patients are young, and the stent will need to stay open and in position (no migration, fracture or thrombosis), for upwards of 50 years. Venous stents have been in existence for a maximum of 35 years; there is no published data with this degree of longevity. Therefore, it is not a trivial decision, and is very different to placing an arterial stent in a 70-year-old arteriopath with at most 15 years of life expectancy.

The final potential indication for venous stenting is postural orthostatic tachycardia syndrome (POTS) and dysautonomia (Ormiston 2022; Knuttinen et al., 2021; Lum et al., 2012)- this is an entirely new and exciting area of research. In a way, it is linked to the realisation that lack of venous return to the right atrium may have profound and unrecognized effects on general well-being (Morris et al., 2020; Smith et al., 2022). It is known that many conditions of unknown cause have been shown to involve impaired orthostatic blood return from the lower body, which may be associated with Ehlers-Danlos syndrome. This may trigger compensatory effects including sympathetic overdrive and high circulating nor-epinephrine, and other problems including interstitial cystitis, chronic bowel problems, migraines, hip pain, excess sweating. A detailed discussion is beyond the scope of this summary, but a cascade of effects may reach many different body systems. There is no evidence that pelvic venous intervention in the absence of pelvic pain is indicated for these other conditions at this time, but this is a possible future research objective (Taylor, 1949; Neĭmark & Shelkovnikova, 2012; Mack et al., 2010; Roma et al., 2018; Chelimsky et al., 2016; Whitehead et al., 2002; Shelkey et al., 2013;  Santoshi 2018).

### The technique of venous stent placement

In contra-distinction to iliac or aortic work, where the common femoral artery is the most common route of access, the common femoral vein should best be avoided in all forms of pelvic venous or ilio-caval venous stent placement. This is because the disease process may often extend down close to the common femoral venous CFV puncture point; the worst-case situation is when a stent should ideally be placed across the actual CFV access point.

For acute iliofemoral deep vein thrombosis, popliteal vein access is preferred. If thrombosed, we employ catheter-directed thrombolysis –or ultrasound-accelerated thrombolysis. If the popliteal vein is open, a single session, mechanical or pharmaco-mechanical thrombectomy may be the best option (O'Sullivan, 2011). Following thrombus removal/dissolution, the proximal stenosis is uncovered; usually, this lesion requires stent placement.

For chronic iliofemoral venous reconstruction or venous stenting as part of pelvic venous disorders, a jugular or mid-femoral route of access is typically chosen. Once the lesion is crossed, and venography/IVUS is performed, balloon angioplasty is required. Typically, we use a 16 mm balloon at high pressure, greater than 20 atm in the common iliac vein and 14 mm in the external iliac vein, and perhaps 12 mm in the common femoral vein. It should be possible to judge which is the dominant inflow in cases of chronic iliofemoral venous occlusion utilising CTV-MRV (Coelho & O'Sullivan, 2019); IVUS will confirm. Following balloon angioplasty, a stent of appropriate length and diameter is chosen; typically, diameters of 16 mm in the common iliac vein, 14 mm in the external iliac vein and common femoral vein are chosen. The stent is positioned to cover the stenosis but avoiding the ostium of the contra-lateral common iliac venous inflow (Bajwa et al., 2019). Stents need to extend from areas with good flow to good flow (normal to normal) (O'Sullivan et al., 2007). Following stent placement, repeat balloon angioplasty is performed again to the nominal diameter of the stent at the same atmospheric pressure as pre-stent placement. If IVUS is available, it is used to confirm that the stent is fully expanded and that there are no synechiae at the inferior end of the stent, particularly if the common femoral venous inflow is compromised (Neglén & Raju, 2002; McLafferty, 2012). Finally, venography is performed, which should demonstrate rapid, in-line flow through the stented segment, with the abolition of collaterals, as confirmation of stent expansion is more accurately assessed by employing intravascular ultrasound.

#### Choice of stent

There are no randomised controlled trials comparing different types of venous stents. The Food and Drug Administration (FDA) mandated trials were set up to evaluate safety and efficacy comparing them with a previous meta-analysis (Razavi et al., 2015). Effectively they were designed to ensure the stents could be commercially available (Murphy et al., 2022; Dake et al., 2021; Hofmann et al., 2023). The physical properties of stents have been evaluated (Dabir et al., 2018); however, at the time of writing, two of the seven stents assessed in that publication are not on the market, and other stents have since received CE mark. Factors to consider include gaps between the interstices so as not to block inflow from side branches (e.g. larger gaps in “Z” stents (Cook Medical, Bloomington, IN, USA), flexibility, degree of foreshortening, radial resistive force etc. No trials exist to compare these, and no recommendations for stent choice in a specific situation are anything beyond personal feeling and experience. There is no evidence that covered stents confer any advantage over bare stents in routine situations.

### Post-operative imaging and follow-up management

Following venous stent placement, MRV is of limited value, as even modern “dedicated” venous Nitinol stents usually contain a small proportion of a ferromagnetic substance which causes signal dropout. Therefore, neither patency nor in-stent restenosis can be identified. Colour Doppler ultrasound (CDUS) is the mainstay for follow-up, but in obese patients, contrast-enhanced CT venography (CTV) may be a reasonable method also. There is marked variation in anticoagulation and antiplatelet management after stenting for non-thrombotic pelvic venous stenosis (Notten et al., 2021).

A recent Delphi consensus among physicians active in venous stenting suggested that anticoagulation is preferred to antiplatelet therapy for the first 6 to 12 months after stenting. At the same time, low-molecular-weight heparin (LMWH) is the first-choice anticoagulant in the first 2 to 6 weeks post-stenting (Milinis et al., 2018). These recommendations may not apply to young female patients at low risk for venous thromboembolism. We typically use LMWH for 2 weeks, followed by a novel oral anticoagulant (NOAC) for 10 weeks. Clinical review at 2 months with CDUS, and symptomatically after that.

Complications of venous stenting (Razavi et al., 2015).

Fracture.

Migration.

Thrombosis.

Rupture.

Fistula formation to adjacent structures.

Acute AV fistula.

Management of these complications is expectant- in other words, complication-specific. Stent fracture did not occur with any degree of frequency in the stent trials (Murphy et al., 2022; Dake et al., 2021; Hofmann et al., 2023). Stent migration is rare if the appropriate diameter and length stent is used (Sayed et al., 2022).

Acute thrombosis is usually managed by venous thrombectomy; chronic stent thrombosis may be more challenging. Rupture and fistula formation both appear to be quite rare unless there has been previous arterial or venous surgery which disrupts the integrity of the surrounding sheath. Acute arterio-venous fistula has been described in conditions where the retroperitoneum is extremely fibrotic.

## Conclusions

Venous stenting is growing in impact in a variety of disease states. Indications include venous stenosis and obstruction, to alleviate pelvic pain, and to improve venous return in disparate conditions. Accurately assessing the clinical significance for any specific degree of degree of obstruction is more challenging than in arteries; and pressure measurements are of little use. This review highlights the importance of this treatment in females.

## Data Availability

Not applicable.

## Abbreviations

CTV: Computed Tomographic venography
MRV: Magnetic Resonance Venography
IV: Intravenous
NaCl: Sodium Chloride
IVUS: IntraVascular UltraSound
Atm: atmospheres (of pressure)
CDUS: Colour Doppler UltraSound
LMWH: Low Molecular Weight Heparin
NOAC: Novel Oral Anticoagulant
NIVL: non thrombotic iliac vein lesion
mm: millimetres
POTS: postural orthostatic tachycardia syndrome
